# A structural equation model looking at student’s participatory behavior and their success in Calculus I

**DOI:** 10.1186/s40594-017-0093-0

**Published:** 2017-11-10

**Authors:** Rachel Elizabeth Keller, Estrella Johnson, Steven DeShong

**Affiliations:** Virginia Polytechnic Institute and State University, Blacksburg, USA

**Keywords:** Calculus, Participation, Success, Structural equation modeling

## Abstract

**Background:**

Government projections in the USA indicate that the country will need a million more science, technology, engineering, and mathematics (STEM) graduates above and beyond those already projected by the year 2022. Of crucial importance to the STEM pipeline is success in Calculus I, without which continuation in a STEM major is not possible. The STEM community at large, and mathematics instructors specifically, need to understand factors that influence and promote success in order to mitigate the alarming attrition trend. Previous work in this area has defined success singularly in terms of grades or persistence; however, these definitions are somewhat limiting and neglect the possible mediating effects of affective constructs like confidence, mindset, and enjoyment on the aforementioned markers of success. Using structural equation modeling, this paper explored the effect of participation on grades in freshman college calculus and investigated whether these effects were mediated by affective variables.

**Results:**

Results indicated that participation had no significant direct effect on any of the success components in the final model—a finding that was not only counterintuitive but actually contradicted previous research done on this data. Participation was however highly correlated with two other exogenous variables indicating it would be inappropriate to dismiss it as being unrelated to success. Furthermore, the results suggested a cluster of affective success components and an achievement component with confidence being the intermediary between the two.

**Conclusions:**

This paper extends upon previous work with this data set in which the effect of participatory behaviors on success was investigated wherein success was measured singularly with expected course grade and affective components of success were not considered. The limited explanatory power of the model, coupled with the seemingly contradictory results, indicates that participatory behaviors alone might be insufficient to capture the complexity of the success response variable.

## Background

In the USA, calculus serves as an introductory course for college freshmen, but especially for those intending to enter into science, technology, engineering, and mathematics (STEM) majors; therefore, student success in Calculus I is of critical importance—without which continuation in a STEM major is impossible. The retention of students in STEM majors has been identified by the President’s Council of Advisors on Science and Technology (PCAST) as a key contributor to the ability of the USA to remain a leader in the STEM fields (Olson and Riordan 2012); PCAST specifically advises that over the next decades, in order to retain our dominance, the nation will require an additional one million STEM majors beyond those currently projected. Currently, fewer than 40% of students who enter college intending to major in science, technology, engineering, or mathematics (STEM) complete a STEM degree (Olson and Riordan 2012), with many students citing introductory math and science classes as a main deterrent (Seymour 2006). With calculus acting as a gatekeeper to a student’s ability to complete an undergraduate STEM degree, post-secondary educators must develop a better understanding of what success in Calculus I looks like and how to help students achieve it.

In 2009, the Mathematical Association of America, through funding from the National Science Foundation, launched its study of Characteristics of Successful Programs in College Calculus^1^ (CSPCC). The research goals of this project were to improve the field’s understanding of Calculus I across the USA and included a large survey of Calculus I students and their instructors. This survey collected data not only on the demographics of the students, but also on their behaviors, perceptions, affect and beliefs about mathematics, and (for a subset) their final grade. This national, and rather large, data set offers a context to investigate relationships between student characteristics and their success in Calculus I. In particular, here, we investigate how a student’s success is influenced by the investment they make in their own educational process (i.e., behaviors both inside and outside of the classroom related to the course).

### Theoretical framework

Success is an inherently difficult construct to measure. For instance, when identifying successful programs in Calculus I, CSPCC looked at, in part, student pass rates, positive affective changes (e.g., in confidence and enjoyment), and intention to take Calculus II (Hsu, Mesa, and The Calculus Case Collective 2014). However, as the name of the project suggests, this was an operationalization of *programmatic success*. For the purposes of this paper, we choose to focus instead on *individual student success* as influenced by *individual* student actions. In doing so, we were informed by a wide variety of definitions and perspectives on success.

In a meta-analysis of over 900 sources, Kuh et al. (2006) sought to synthesize relevant literature and broadly define student success. Their definition reads as follows: “Student success is defined as academic achievement, engagement in educationally purposeful activities, satisfaction, acquisition of desired knowledge, skills, and competencies, persistence, attainment of educational outcomes, and post-college performance” (p. 5). The proliferation of studies concerned with identifying constructs that promote academic success has done the field no favors in terms of clarifying the definition; on the contrary, it has become a catchall phrase. “An amorphous construct that broadly incorporates a broad range of educational outcomes from degree attainment to moral development” (York et al. 2015, p. 1), the definition seems to be ever expanding to the extent that an inverse relationship now exists between the length of the definition and its usefulness. The meta-analysis results suggest that rather than continually refining the definition for succinctness, the field has chosen instead to amplify the definition to include any and all individual measures that have been proposed.

The purpose of the present study is to find middle ground between the multitude of studies for which success was measured by an individual achievement measure or by proxy (i.e., persistence) and attempting to measure and/or define all theorized components and the relations therein. By drawing on the large CSPCC data set, our intention is to streamline Kuh et al.’s (2006) definition, restricting our attention to those components that we believe are reasonably addressed by the CSPCC survey items and have been historically linked to success in mathematics. Specifically, we will consider confidence, enjoyment, mindset, and achievement. Achievement (as measured by course grade) is certainly a necessary factor of success, as students cannot continue perusing a STEM degree without a passing grade in Calculus I. However, we argue that while achievement is imperative for success, it is by no means sufficient. For instance, Rasmussen and Ellis (2013) found that many students choose to not continue onto Calculus II (and therefore leave STEM majors) despite having the grades necessary to do so.

In mathematics, many researchers have looked at student affective features when studying student success. For instance, as summarized by Hall and Ponton (2005), the research findings in collegiate development mathematics report that “academic self-concepts, attitudes toward success in mathematics, confidence in ability to learn mathematics, mathematics anxiety, self-efficacy, and locus of control are all variables that affect student goals, performances, and attainments in mathematics” (p. 26). The importance of these affective components are not limited to developmental mathematics and are often discussed in relation to student success in Calculus I (e.g., Pyzdrowski et al. 2013). However, instead of considering these as indicators for success, in keeping with Kuh et al.’s (2006) definition, we choose for our conceptualization of success to include student reports of confidence, enjoyment, and student mindset in relation to experiencing difficulty while learning mathematics. Once an operationalized construct for success is derived, we will then investigate the influence of student participation on success and the mediated effects therein.

Multiple authors have found a correlation between participation and achievement. For instance, there has been research indicating that homework completion (e.g., Vestal 2008) and participation in classroom discussion (e.g., Lucas 2009; Rasmussen and Ellis 2013; Keller et al. 2016) are of critical importance for student success. That being said, when considering student participation, we must be aware that this characterization extends beyond traditional notions of participation and recognize that participatory behaviors manifest themselves in a variety of ways, both inside the classroom and outside of it. As an example, tutoring centers and office hours are a nearly universal support offered to calculus students and, when utilized, students often point to these as valuable resources (Johnson and Hanson, 2015). Thus, following the establishment of our success construct, we will then look for direct and indirect effects of a variety of student participatory behaviors (e.g., attending office hours, doing homework, contributing to class discussions) on student success. We are motivated to look for evidence of a causal relationship between participation and the affective components of success by Muis (2004) who stated that, “researchers are not capable at this point of making a cause-and-effect claim that students’ classroom experiences greatly influence their beliefs, but the empirical evidence does correlate with such a claim” (p.338).

## Methods

### Data source and sample

The present study is situated within the larger research project, CSPCC, designed to gain a nationwide overview of college calculus programs. The CSPCC project^2^ used a stratified random sample of colleges and universities in the USA based on the highest degree granted at each university (Associate’s, Bachelor’s, Master’s, or Ph.D.). This research was of a hierarchical nature (course coordinators, instructors, and students) and invoked repeated sampling (start of term, end of term, and follow-up). For the purposes of the present study, we limited our data set to those student respondents who had completed the end of semester survey and for whom the final course grade had been instructor verified (*n* = 1208). It was not the intention of this research to investigate invariance of the measurement or structural model with respect to gender, race, or other similar classifications; thus, descriptive statistics concerning demographic information were not computed.^3^

### Building the measurement model

The planned methodological approach for building the measurement model was to propose a factor solution using exploratory factor analysis (EFA) and then cross-validate the model using confirmatory factor analysis (CFA). To that end, the data set was randomly divided into calibration (40%) and validation (60%) subsamples (using IBM SPSS 23). Rather than propose a factor loading structure a priori, we selected items from the CSPCC Student End Survey that in our estimation measured or were in some way indicative of the larger constructs under investigation. Using the calibration sample, the data’s suitability for factor analysis was checked, an EFA was run on each set of items, and an initial model was proposed.

To measure success, we included seven Likert-type items from the Student End of Term survey that we felt supported the idea of success as a multi-faceted construct. These items included self-reported indications of confidence, enjoyment, and mindset (as captured by items that pertain to students’ attitudes towards experiencing challenges) as well as an achievement measure (final course grade). (See Table 1 for survey items.) It is important to note that the achievement measure was *not* self-report data. Since the students were asked to indicate their *expected* course grade (before grades were released), these reports were of questionable accuracy; therefore, grade information was obtained at the department level after the semester ended and matched with student ID numbers. Alphabetic grade codes were recoded numerically on a 4.0 scale by the research team. Participation was measured with a list of eight Likert-type items from the Student End survey that investigated in-class behavior and out-of-class preparation. These items included questions about how the students participated in class, such as contributing to discussions, asking questions, and actively following the lecture; and help-seeking behaviors, such as visiting office hours, and getting help from tutors. (Again, see Table 1 for survey items.)

**Table 1:** Survey items and latent variables

Item	Wording	Scale	Latent variable	Cronbach’s *α*
Q6b	I am confident in my mathematical abilities.	1 = strongly disagree to 6 = strongly agree	Confidence	0.856
Q6c	I am good at computing derivatives and integrals.			
Q6d	I am able to use ideas of calculus (e.g. differentiation, integration) to solve word problems that I have not seen before.			
Q6f	I enjoy doing mathematics.	1 = strongly disagree to 6 = strongly agree	Enjoyment	0.80
Q12	If I had a choice: A = I would never take another mathematics course, B = I would continue to take mathematics.	1 = completely A, 2 = more A than B, 3 = more B than A, 4 = completely B		
Q8	When experiencing a difficulty in my math class: A = I try hard to figure it out on my own, B = I quickly seek help or give up trying.	1 = completely A, 2 = more A than B, 3 = more B than A, 4 = completely B	Mindset	0.497
Q9	For me, making unsuccessful attempts when solving a mathematics problem is: A = a natural part of the problem, B = an indication of my weakness is mathematics			
GPA	Instructor-verified final course grade recorded numerically	0.0 to 4.0	Achievement	N/A
Q33a	During class, I contributed to discussion.	1 = never to 5 = every class session	Participation	0.841
Q33c	During class, I asked questions.			
Q33b	During class, I was lost and unable to follow the lecture.	1 = never to 5 = every class session	Difficulty	0.504
Q33d	During class, I simply copied whatever was written on the board.			
Q34b	How often did I visit my instructor’s office hours?	1 = never to 5 = more than once/week	HelpSeeking	0.505
Q34c	How often did I use online tutoring?			
Q34d	How often did I visit a tutor to assist with the course?			
Q35d	I completed all my assigned homework.	1 = strongly disagree to 6 = strongly agree	Homework	N/A

*N/A* not applicable

Since the primary methodological technique used in building the measurement model was to be factor analysis, it was necessary to test the data for suitability with regard to the underlying assumptions. The sample size was well above recommended minimum guidelines (at least 10 subjects/factor) since we only wished to extract a few factors. Missing data was not problematic for our data and was handled by listwise deletion since the sample size was large and the percentage of missing data was so small^4^ (< 2%).

Measures of skewness and kurtosis were computed (using IBM SPSS 23), along with means and standard deviations, for all of the items. The skewness and kurtosis values were divided by their standard errors to determine their severity; values > ± 3.25 indicated significant severity. Both skewness and kurtosis were a problem for a majority of these items (which is to be expected with ordinal data). These issues could have been corrected with a transformation, but one was not performed at this time due to the exploratory nature of this analysis.

The univariate normality assumption was checked with the one-sample Kolmogorov-Smirnov Test (SPSS) and multivariate normality was assessed with the Shapiro-Wilk goodness-of-fit test (using JMP Pro 11) on the distribution of the Mahalanobis distances. There were significant departures from normality on both tests.

With regard to outliers, multivariate outliers have more influence on the factor solution than do univariate outliers, so that is what we checked. Using the Mahalanobis distance values, multivariate outliers can be identified using the *χ* ^2^ distribution. None were identified.

Factor analysis assumes the absence of multicollinearity and singularity. To check for this, variance inflation factors (VIFs) were computed (using SPSS). The values were reasonably low (< 10); therefore, multicollinearity is not a problem.

The final diagnostic check for the suitability of the data for factor analysis was the factorability of *R*. Low bivariate correlations (< .30) indicate that factor analysis could be inappropriate for the given data. This assumption was reasonably met for our data.

While the data did demonstrate significant departures from normality (as is expected with ordinal data), the other diagnostic checks were appropriately met and so we concluded that our data was suitable for factor analysis. For cross-validation purposes, it was necessary to split the data set into a calibration sample and a validation sample. It was assumed that both subsamples of the data would display similar properties with regard to normality, skewness, kurtosis, factorability, etc., and therefore, it was not necessary to analyze the validation sample with the same scrutiny as was done for the calibration sample.

For both the success and participation items, we hypothesized a four-factor solution based on theory. We initially extracted this number and confirmed with the screeplot and eigenvalues. An oblique rotation was initially proposed until confirmation of applicability of an orthogonal rotation could be obtained; the factor correlation matrix verified the appropriateness of the oblique rotation in both cases. The generalized least squares extraction method was used in favor of maximum likelihood because of the lack of normality of the data. Small factor loadings (< .30) were suppressed.

In both cases, the factor solution was quite satisfactory. The Kaiser-Meyer-Olkin measure of sampling adequacy was above the recommended value of .60 (.833 and .636 respectively) and Bartlett’s test of sphericity was significant (significant (*χ* ^2^ (28) = 1468.46, *p* < .001; *χ* ^2^ (28) = 784.942, *p* < .001); collectively, these measures suggest reasonable factorability and indeed that was demonstrated.

The items were generally well defined by the four-factor solution: none of our variables failed to load onto any factor (i.e., loadings < .30). A clean, simple structure (no cross-loading) was observed with the four-factor solution in both cases. Total variance explained by these solutions was acceptable (64.68 and 56.44%, respectively).

Cronbach’s alpha coefficients were computed and were mostly satisfactory, especially given the exploratory nature of this study analyzing extant data (see Table 1). Interpretive labels for the factors were assigned in an effort to indicate the underlying similarities between survey items that loaded together. For instance, Q8 and Q9 both address student attitudes towards facing challenges in the learning process. Such attitudes, suggesting that students either view struggle as part of the learning process or that struggle is a sign that they are unable to succeed in mathematics, are related to Dweck’s (2006) notions of mindset. While we are not claiming that our two items are a perfect measure for student mindset, especially given the mediocre Cronbach’s alpha, we did feel that the mindset construct warranted inclusion. As discussed by Boaler (2013), Dweck’s work has had “one of the biggest impacts on education of any research volume ever published” (p. 143). Thus, by retaining this variable in our work, we hope to use our exploratory analysis to generate hypotheses for further research. Other interpretive labels include participation, to capture those items about how students verbally participated in class; and difficulty, to capture those items that illustrated students’ struggles to interact with the in-class presentation of the material in real time.

It is important to note here that while the number of indicators per factor might appear to be insufficient, this was a conscious choice that we feel is both theoretically and methodologically defensible.^5^ In typical factor analysis, the primary objective is dimension reduction in which fewer factors with numerous indicators are preferable to many factors with few indicators. In the present study, the objective was to build and characterize latent constructs (based on theory) that could inform the measurement and structural models—a distinction that supports the decision to retain factors with few indicators. As a specific example, one might argue that the participation and difficulty factors should be merged into a single construct with four indicators on the basis that all four items address in-class activity on the part of the student; however, we contend that the nature of the behaviors is not only distinct enough to warrant separation but, in doing so, provides the opportunity to explore additional relationships in the path model. As explained by Hayduk and Littvay (2012), “multiple indicators hamper theory by unnecessarily restricting the number of modeled latents” which is undesirable because “additional latent variables permit stronger statistical control of potential confounders, and encourage detailed investigation of mediating causal mechanisms” (p. 1).

### Building the structural model

**Fig. 1:**
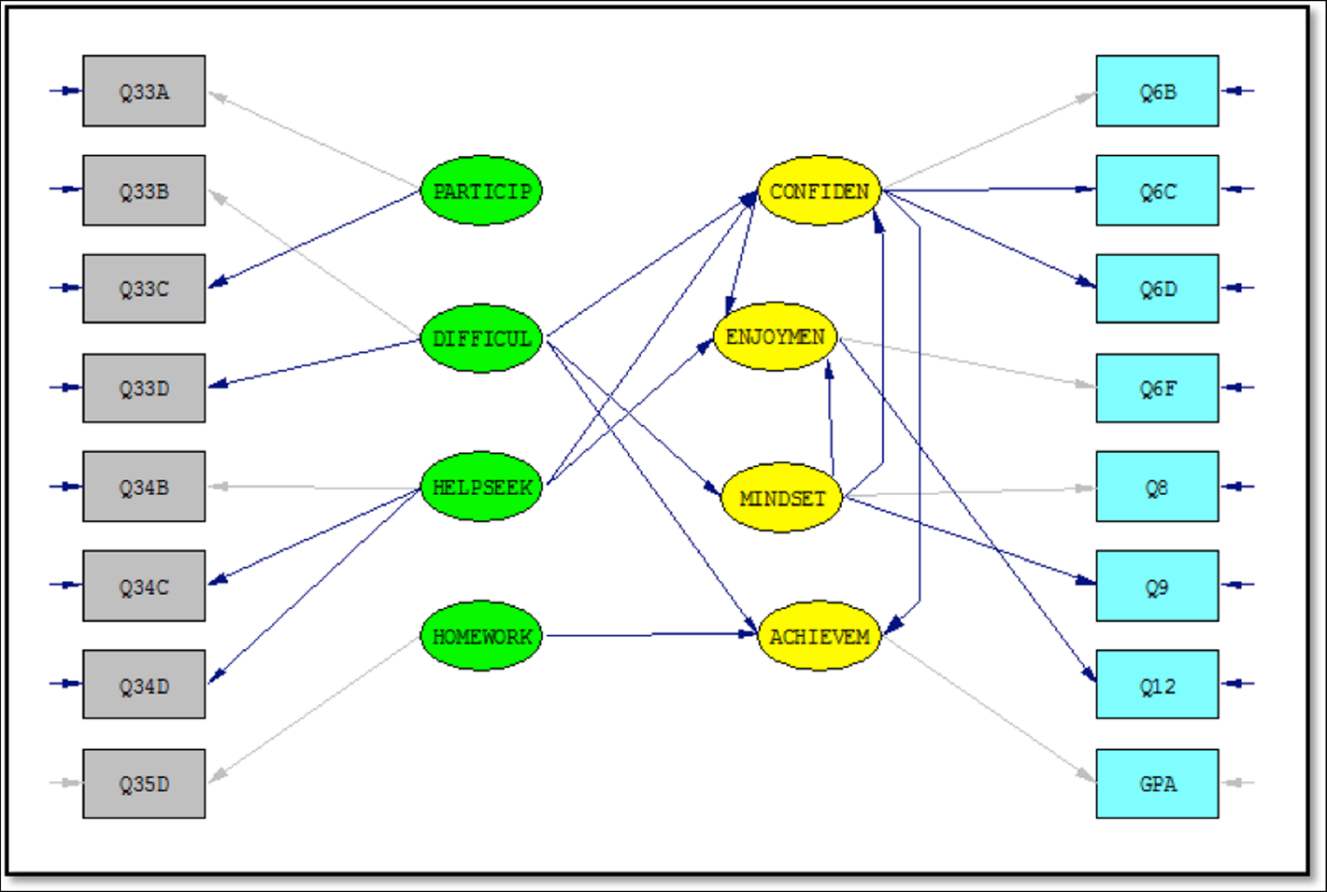
Hypothesized structural model

The exploratory nature of this research allowed us to take a somewhat data-driven approach to the structural model. Using the proposed measurement model from the EFA, we started with an initial structural model that included a full Phi matrix (exogenous factor correlations), a full Gamma matrix (paths from exogenous to endogenous variables), and a full Beta matrix (paths from endogenous to endogenous variables) run (using LISREL 9.2) on the calibration sample using the default estimation method of maximum likelihood.^6^ Using the modification indices as a guide and eliminating insignificant path coefficients, the model was reduced step-wise until only significant paths remained. This generated our hypothesized final model that would be subject to cross-validation. This can be seen in Fig. 1. Reasonably good fit indices resulted: *χ* ^2^(92) = 239.43, *p* < .0001,

goodness-of-fit index (GFI) = .941, standardized root mean square residual (RMR) = .0552, root mean square error of approximation (RMSEA) = .059, parsimony normed fit index (PNFI) = .695, comparative fit index (CFI) = .94; thus supporting the validity of the proposed model. Looking beyond the usual measures of fit and adequacy, residual analysis revealed nothing out of the ordinary and tests of parameter coefficients significance all looked satisfactory.

### Validating the model

We cross-validated our model through the use of invariance testing. In this procedure, the freely estimated parameters of the validation sample are constrained to equal the estimates from the calibration sample and a chi-square difference test is performed. A lack of statistical significance would indicate that those parameter estimates are invariant across the groups; that hypothesis was upheld in this case for the most part (see Table 2). The cross-validation was done in two steps in order to test the measurement and structural parts of the model separately. Invariance of the factor loadings, indirect paths, and direct paths was upheld. The only questionable validation occurred when testing the factor correlations. This will be examined in greater detail in the final solution.

**Table 2:** Cross-validation results

Model	*χ* ^2^	df	RMSEA	Δχ^2^	Δdf	*p*
Unconstrained model	661.79	184	0.062			
Measurement model constrained (LX/LY)	668.17	192	0.06	6.38	8	.5 < *p* < .75
Structural paths constrained (GA/BE)	681.28	202	0.059	19.49	18	.25 < *p* < .5
Factor correlations constrained (PH)	695.8	206	0.059	34.01	22	.01 < *p* < .05

### Final model assessment

The proposed model from the cross-validation procedure was fitted with the data set as a whole to assess adequacy and goodness-of-fit. Standard deviations and interitem correlations can be seen in Table 3. Reasonably good fit indices resulted: χ^2^(92) = 440.92, *p* < .0001, GFI = .955, standardized RMR = .0568, RMSEA = .0575, PNFI = .709, CFI = .94; thus supporting the validity of the proposed model. Looking beyond the usual measures of fit and adequacy, residuals analysis revealed nothing out of the ordinary and all parameter estimates were significant (|*t*| > 2.5).

**Table 3:** Item correlation matrix

Item	1	2	3	4	5	6	7	8	9	10	11	12	13	14	15	16
Q33a	1.0															
Q33b	− .13	1.0														
Q33c	.72	.045	1.0													
Q33d	− .217	.346	− .134	1.0												
Q34b	.237	.094	.314	.03	1.0											
Q34c	− .057	.184	.071	.119	.097	1.0										
Q34d	− .019	.329	.074	.176	.205	.298	1.0									
Q35d	.086	− .134	.022	− .033	.058	− .014	.015	1.0								
Q6b	.197	− .416	.065	− .306	− .101	− .177	− .266	.204	1.0							
Q6c	.193	− .351	.092	− .245	− .094	− .155	− .254	.204	.711	1.0						
Q6d	.197	− .357	.088	− .315	− .045	− .061	− .236	.177	.661	.64	1.0					
Q6f	.149	− .285	.044	− .161	− .021	− .103	− .22	.12	.584	.443	.44	1.0				
Q8	− .04	.252	.068	.223	.136	.073	.256	− .087	−.32	− .284	− .304	− .314	1.0			
Q9	− .085	.237	− .043	.162	.047	.072	.113	− .067	− .346	− .264	− .274	− .302	.372	1.0		
Q12	.192	− .282	.086	− .174	−.02	− .084	− .157	.126	.504	.363	.378	.684	− .282	− .279	1.0	
GPA	.157	− .323	− .006	− .237	− .094	− .189	− .284	.255	.455	.418	.396	.285	− .303	− .215	.277	1.0
SD	1.26	1.005	1.14	1.43	1.194	1.203	1.37	1.35	1.26	1.035	1.233	1.379	0.815	0.913	1.076	1.062

**Fig. 2:**
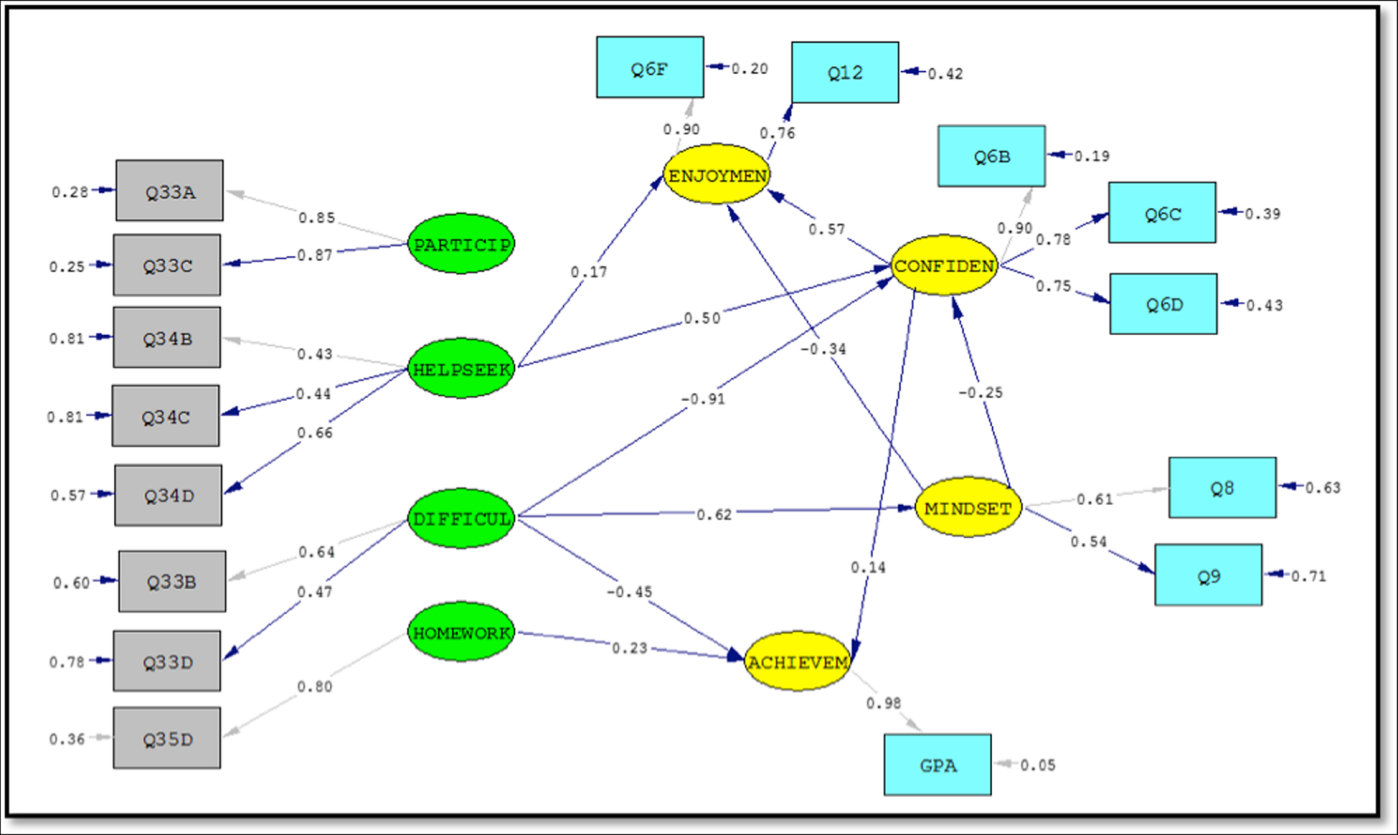
Standardized solution

The cross-validation procedure suggested that the factor correlation estimates were likely invariant across the calibration and validation samples, specifically the correlation from HelpSeeking Behavior to Difficulty. In the full model, the Phi matrix was examined for insignificant parameter estimates and modification indices. There was no compelling evidence to justify alteration of this part of the structural model, and thus, the model remained unaltered from the proposed state. The standardized solution can be seen in Fig. 2.

## Results

This research estimated a model of how a student’s behavior, both in and out of the classroom, affects his/her success in calculus whereby success was defined as a multi-faceted construct comprised of Enjoyment, Confidence, Mindset, and Achievement. Assessment of the global fit indices indicated that the model was adequate enough to warrant interpretation of both the measurement and structural parts of the model.

### Measurement model

The role of the measurement model is to specify the relationship between a latent variable and its indicators. Significant factor loadings and high reliabilities are desired, as well as high squared multiple correlations. All of the factor loadings in this model were significant; however, not all items were collectively reliable measures of the construct under investigation.

Looking at the exogenous variables, Participation was sufficiently reliable (*α* = .841); but the same could not be said for Difficulty and HelpSeeking (*α* = .504, *α* = .505 respectively). Low reliabilities indicate that these items are perhaps not measuring the same underlying latent construct or at the very least are not being interpreted by the respondents in a similar fashion. This is not surprising for Difficulty because *simply copying notes* could be an indication of apathy as much as confusion and the item is somewhat ambiguous for capturing students who are truly experiencing difficulty with the material. On the other hand, the low reliability for HelpSeeking is surprising because attending office hours and using a tutor would seem to be actions dictating similar circumstances.

Looking at the endogenous variables, we see that Confidence (*α* = .856) and Enjoyment (*α* = .80) were quite satisfactory and that MindSet, as previously discussed, was of questionable reliability (*α* = .497). Unfortunately, this was a complication we had anticipated, but one that did not have an immediate solution. Capturing a person’s location on the growth/fixed mindset continuum is difficult under the best of circumstances, but certainly when using items not necessarily designed for that purpose. The research-supported (Dweck 2006) items were not available for this study, and the research team chose the items that we felt best embodied the spirit of the construct under investigation because we felt it was too important an idea to exclude from the model on the basis of potentially questionable items. By retaining the, admittedly not ideal, Mindset construct in the model, we are able to gain insight into how mindset may be related to other components of success. Thus, we see these results as preliminary, generating hypotheses rather than firm conclusions.

The squared multiple correlations indicate the percentage of variation in a latent variable that is being explained by the model. Our model was best able to explain Confidence, followed by Mindset, Achievement, and lastly, Enjoyment. 53.6% of the variation in Confidence was accounted for by Difficulty, HelpSeeking, and Mindset. 38.6% of the variation in Mindset was accounted for by Difficulty. 36.1% of the variation in Achievement was accounted for by Difficulty, HelpSeeking, Homework, Confidence, and Mindset. 28.2% of the variation in Enjoyment was accounted for by Difficulty, HelpSeeking, Confidence, and Mindset.

It is important to keep in mind that low squared multiple correlations do not necessarily indicate a poor model, rather, an incomplete one. When considering any latent variable, it is possible that the selected indicators are necessary but not sufficient to fully explain the variance of that construct. In the case of Achievement for instance, it is reasonable to assume that any or all of the following—prior knowledge, past experience, instructor actions, demographic factors—would influence course grades and that student participatory behaviors alone would only represent a partial explanation. Identifying and measuring the missing indicators is a topic for future research and beyond the scope of the present study. Another possible explanation for low squared multiple correlations is not a lack of factors like in the previous example, but rather a lack of high-quality items per factor (or simply a lack of items); the use of extant data in this study certainly lends credibility for this explanation.

### Structural model

Moving from the measurement part of the model to the structural part, there are three types of parameters of interest. The Phi matrix describes the relationship of the exogenous variables to each other. The Gamma matrix describes the direct effects of the exogenous variables on the endogenous variables. The Beta matrix describes the relationship of the endogenous variables to each other.

Due to the nature of the exogenous items (i.e., all items represented an investment on the part of the student in terms of time or attention), significant factor correlations were expected. Only two of the six potential correlations were dropped from the model for insignificance: Difficulty with Participation and Difficulty with Homework. Of those remaining, it was somewhat unsurprising that the most significant path was seen between Difficulty and HelpSeeking.

Moving to the relationship between the student behaviors and the success components, we note several interesting findings. First, Participation had no significant direct effect on any of the success components. This was certainly surprising since it runs counter to previous research (Keller et al. 2016) that indicates that increased levels of in-class participation are directly correlated with higher grades. HelpSeeking behaviors had a significant effect on both Confidence and Enjoyment, but neither Mindset nor Achievement. The lack of an effect on Achievement, while possibly counterintuitive, is not necessarily without reasonable explanation. Perhaps it is the timing of the help-seeking behavior that is confounding the situation. If students are waiting until they are already failing the course to seek help, these last-ditch efforts are unlikely to yield significant improvement in final course grades and would then understandably have little effect on achievement. Difficulty had a significant effect on Mindset and (unsurprisingly) a negative effect on both Confidence and Achievement. Surprising here was the lack of direct effect on Enjoyment. It would seem that those experiencing difficulty in class would be less likely to be enjoying the course, but that insinuation is only evidenced in an indirect effect (through Confidence and Mindset) in this model. Homework had a significant direct effect on Achievement, but failed to have any effect—direct or indirect—on any other component of success.

In addition to considering the effects of the student behavior variables on success, this research was also interested in identifying relationships between the components of success, and specifically which, if any, had a direct effect on achievement. Enjoyment had no effect on Mindset, Confidence, or Achievement. Mindset had a significant effect on both Confidence and Enjoyment which is unsurprising. Students with a growth mindset are more likely to expect incorrect answers and temporary failures as an inevitable part of the mathematical process; therefore, not attributing these setbacks to a lack of ability renders these students less likely to be discouraged and/or doubt their potential to succeed in the course relative to fixed mindset classmates. Confidence had a direct effect on Achievement and was the mechanism for the indirect effects on Achievement stemming from Difficulty and HelpSeeking. A complete summary of (standardized) direct, indirect, and total effects can be seen in Table 4.

**Table 4:** Summary of latent variable effects

	Difficulty			HelpSeeking			Homework			Confidence			Mindset		
	Direct	Indirect	Total	Direct	Indirect	Total	Direct	Indirect	Total	Direct	Indirect	Total	Direct	Indirect	Total
Confidence	*−* .*91*	− .156	− 1.066	.501		.501							*−* .*251*		*−* .*251*
Enjoyment		− .819	− .819	.174	.287	.461				.*573*		*.573*	*−* .*335*	*−* .*144*	*−* .*479*
Mindset	.*621*		.621												
Achievement	*−* .*451*	− .151	− .602		.071	.071	.233		.*233*	.*142*		.*142*		*−* .*036*	*−* .*036*

## Discussion

For this model, we chose to focus on eight survey responses, all pertaining to students’ participatory behavior. Remarkably, these eight questions (from an extant data set that we did not collect with this model in mind) were able to account for a significant amount of the variance of our latent construct (i.e., our four-faceted operationalization of success); thus, the participatory behaviors captured by these eight questions are clearly important for student success. In fact, these behaviors alone, in the absence of data on student demographics and classroom experiences, were able to account for roughly a third of the variation for each of the four endogenous variables (53.6% of the variation in Confidence was explained, 38.6% of the variation in Mindset was explained, 36.1% of the variation in Achievement was explained, and 28.2% of the variation in Enjoyment was explained.). The purpose of this study, however, was not limited to merely explaining variation in success based on student behaviors, but to identify and explain mediating relationships among the components of success. To that end, we wish to highlight and elaborate on three areas of our results: the relationships between the exogenous variables, the relationships between the endogenous variables, and the relationships between the two sets.

First, in regard to the exogenous variables, we found that Participation in discussion and lecture (i.e., through asking questions) had no significant direct effect on any of the success components in the final model (Fig. 2). This was not only counterintuitive; it actually contradicts previous research done on this data that found a relationship between student reports of in-class participation and expected final grades (Keller et al. 2016). In this previous study, contributing to class discussions was a significant predictor in the logistic model for success (as designated by pass/fail) with successful students, on average, contributing to discussion more frequently than unsuccessful students. However, it is important to note that Participation was highly correlated to two of the other exogenous variables, Homework and HelpSeeking. Thus, while no direct effects were found, this variable is an important piece of the measurement model and it would be inappropriate to dismiss it as being unrelated to success. (Incidentally, attempts to remove this latent construct altogether resulted in decreased fit statistics and invalidation of significant path coefficients.)

Second, we note that the only direct effect between the other three endogenous variables and Achievement was through Confidence. This supports our argument for not considering Achievement as the only measure of success. In fact, we see that both Confidence and MindSet have direct paths to Enjoyment, with no direct paths originating from Enjoyment. This suggests a cluster of affective success components and a grade component, with confidence being the intermediary between these two. This is particularly important in regards to Calculus I students—where many students choose to not continue onto Calculus II (and therefore leave STEM majors) despite having the grades necessary to do so (Rasmussen and Ellis 2013). Not only is considering final course grade insufficient for capturing success, there are ways to be successful that do not have direct effects on grades.

Finally, when looking at the relationships between the exogenous and endogenous variables, we note that many theoretically supported effects were not found. In our model, we found no direct or indirect effects from homework to confidence, while Hutchison et al. (2006) found that first year engineering students “cited their ability to complete assignments as influencing their efficacy beliefs” (p. 43). Additionally, we found no effects (direct or indirect) between help-seeking behavior and our operationalization of mindset, even though seeking help from others is taken to be evidence of a resilient mindset (Yeager and Dweck 2012). However, this may be due to the questionably problematic nature of the MindSet variable in our model. Carefully designed studies would be needed to further investigate these relationships.

Also, as previously mentioned, Participation had no effect on any dimension of success, which is counter to previous findings on this same data set (Keller et al. 2016; Rasmussen and Ellis 2013). A possible explanation to this might be found in the modification indices which suggested that the model fit might be improved if item 34b (visiting instructor’s office hours) was an indicator for the latent construct Participation. While it was our intention for Participation to measure the frequency and quality of in-class contributions on the part of the student, this modification suggests that perhaps this collection of items is, in fact, measuring the willingness or ability of the students to interact with the instructor—something that is as much a commentary on the instructor’s demeanor and pedagogical style as it is on the inclination of the student to be active and engaged in the classroom. This suggests that future research should look at how success is affected when both student and instructor characteristics are considered simultaneously and the mediating effects therein.

## Conclusions

The objective of this research was to examine how a student’s investment in his/her education would affect success. Exploratory in nature, this research was not trying to corroborate or invalidate previous research linking participation and academic success and/or achievement; rather, the purpose was to explore existing data in a novel way. The scale and scope of the CSPCC project has generated the production of a sizeable collection of analyses and papers. Not wishing to be derivative (pun intended!), this work sought to define and measure success as a multi-faceted construct and not as a unidimensional measure of grades or persistence or disposition alone.

There are two main limitations of this study that we would like to highlight. First, while the use of national data lends credibility to our findings and provides samples numerous enough to permit cross-validation, the information comes in the form of extant data. Not being privy to the instrument-design process, we were not able to craft the items in a manner consistent with our research goals and theoretical framework; thus, we were faced with a paucity of items relevant to particular constructs (e.g., achievement, homework) and potentially unreliable indicators for others (e.g., mindset). Future research in this area could build upon this work with a revised instrument that accounts for these issues. The other main limitation of this work is the lack of comprehensiveness. This model is only able to explain between 28 and 56% of the variation in success (by component) indicating that participatory behaviors alone are insufficient to capture the complexity of this response; therefore, we stipulate that future research in this area should include variables that encompass prior knowledge, past experience, teaching practices, and demographic factors in an attempt to supply the information necessary to explain the as yet unaccounted for variation in success. That being said, while we acknowledge that external influences do exist (i.e., teaching practices), we contend that each student’s achievement is ultimately the result of his/her own actions within the course and students must share in the responsibility for their success. Thus, we see this work not as a forest with missing trees, but rather as a close, careful examination of a single tree within the larger forest that has merit in and of itself.

Despite the aforementioned limitations, we feel that this research is not without merit. The use of national data lends credibility to the results. The significance of the path coefficients and the magnitude of the effects (in the final model) justify the importance of these factors in measuring success in Calculus I; furthermore, the successful cross-validation supports the robustness of the theoretical model. The use of structural equation modeling allows the specification of both direct and indirect effects—a mediated effects model—which is more robust and better reflects the complexity of the relationships among the factors as compared with models with only direct effects (Singh et al. 2002). In that way, the present study has extended the previous work (using the same data) of (Keller et al. 2016) in which the effect of participatory behaviors on success was investigated wherein success was measured singularly with expected course grade and affective components of success were not considered.

Understanding the factors that influence a student’s decision to persist in a STEM major is of critical importance for arresting the attrition that currently plagues our STEM-intending undergraduates. While we acknowledge that this is but a partial explanation, we offer this work in the hopes that it will shed light on this important issue based on the premise that success in calculus is non-negotiable for the completion of a STEM degree.

## Abbreviations

CFA: Confirmatory factor analysis
CSPCC: Characteristics of Successful Programs in College Calculus
EFA: Exploratory factor analysis
PCAST: President’s Council of Advisors on Science and Technology
STEM: Science, technology, engineering, and mathematics

## Footnotes

1 NSF DRL #0910240

2 For further details, see the *MAA Notes* volume *Insights and Recommendations from the MAA National Study of College Calculus* (Bressoud et al. 2015) or visit the website at www.maa.org/cspcc).

3 This information has been retained and will be considered in future research.

4 According to Tabachnick and Fidell (2013), “If only a few data points, say 5% or less, are missing in a random pattern from a large data set, the problems are less serious and almost any procedure for handling missing values yields similar results. Unfortunately, there are as yet no firm guidelines for how much missing data can be tolerated for a sample of a given size” (p. 63).

5 Structural equation modeling developed as a statistical melding of path analysis and factor analysis that obscured a fundamental tension between a factor preference for multiple indicators and path modeling’s openness to fewer indicators. One or two indicators are often sufficient, but three indicators may occasionally be helpful. More than three indicators are rarely warranted because additional redundant indicators provide less research benefit than single indicators of additional latent variables. Scales created from multiple indicators can introduce additional problems, and are prone to being less desirable than either single or multiple indicators. (Hayduk and Littvay 2012, p.1)

6 We acknowledge that violation of the normality assumption calls into question the validity of using the ML approach; however, it is known that use of this method will not change the factor structure or loadings and will only affect the standard errors. To compensate for this, we advise that interpretation of parameter tests of significance should be done with extreme caution.
